# Trial of Robert Pate for the Outrage upon Her Majesty

**Published:** 1850-10-01

**Authors:** 


### TRIAL OF ROBERT PATE FOR THE OUTRAGE UPON HER MAJESTY.

The prisoner was arraigned at the Central Criminal Court. The learned judges were, Mr. Baron Alderson, Mr. Justice Patteson, and Mr. Justice Talfourd.

Upon the prisoner being placed at the bar,

Mr. Clerk read the indictment, which, in the first count, charged the prisoner with having with a certain offensive weapon, that is to say, a stick, unlawfully and maliciously struck at the person of our Lady the Queen, with intent to injure the person of our Lady the Queen.

The prisoner pleaded "Not guilty" in a loud tone, and the jury were then impanelled.

The Attorney-General opened the case. When he considered, on the one hand, that the object of the attack of the prisoner was a lady and a sovereign who had endeared herself to her subjects by her great virtues, and that, on the other, the person charged with the commission of the offence filled the position of a gentleman, and a man of education, and who had also at one time held her Majesty's commission, he could not but feel that these were circumstances which very greatly aggravated the offence imputed to the prisoner. The prisoner was the son of a gentleman of fortune and station residing at Wisbeach, and who had filled the office of high sheriff for the county of Cambridge, and the prisoner had for some years been cornet and lieutenant in the 10th Hussars. For some time, however, he had retired from the army, and it would appear, that after this he had resided in London, and for a considerable time had led a life of complete quiet and retirement. The learned Attorney-General then proceeded very briefly to state the circumstances of the assault upon her Majesty, and said these were the facts upon which the charge was preferred against the prisoner, and he had no doubt he should establish them clearly by the evidence of one or two witnesses. He went on to say that it was not for him to speculate as to the motives that had led to the commission of the act—motives might be suggested, but he did not think it would be discreet to do so. He could not tell what excuse was to be offered for the prisoner, but he had heard that the unfortunate gentlemen, or his friends acting for him, intended to endeavour to establish that he was not in such a state of mind as to render him accountable for his actions. If this should turn out to be the case, the jury would permit him to suggest that they ought not to give effect to such a defence unless it was made out by strict and complete proof, and that they should not permit the law to be evaded upon slight grounds.

The following witnesses were then examined:—

Colonel Grey, examined by the Solicitor-General—"I hold the office of Equerry to her Majesty. I remember her Majesty leaving Buckingham Palace on Thursday, the 27th of June, in an open barouche. The carriage drove to Cambridge House, where her Majesty alighted. She returned to the carriage about half-past six, going out by the east gate. Several persons had assembled outside. The carriage went out of the gate very slowly, and at the same moment I saw a well-dressed man step up to the carriage, and immediately afterwards he was seized by the footman. That man was the prisoner. He was taken into custody immediately. When her Majesty arrived at Buckingham Palace, Sir James Clark was sent for, and I saw her Majesty's head bandaged, and blood coming through the bandage."

Robert Renwick said—"I am serjeant footman to her Majesty. I remember her Majesty going to Cambridge House on the 27th of June. I was sitting behind the carriage when it came out of the gate of Cambridge House. I saw the prisoner strike the Queen with a small cane on the forehead. I seized him immediately. The crowd closed upon him, and he was secured." By Mr. Cockburn—"He was roughly handled by the crowd."

The cane was produced and identified by the witness.

James Silver—"I am serjeant of the A division of police. I saw her Majesty's carriage leave Cambridge House on the evening in question, and hearing an exclamation from the crowd, I looked towards them, and saw the prisoner with the stick in his hand, which I now produce. The crowd were very indignant, and it was with difficulty I could preserve him from their violence. The prisoner gave his name at the station-house as Robert Pate, and said he was formerly lieutenant in the 10th Hussars. He cautioned some of the witnesses who were being examined, and said they did not know whether he hit at the Queen's bonnet or her head, and added that it was a slight blow with a light stick."

Samuel Cowling said—"On the evening of the 27th of June, I was among the crowd standing in front of Cambridge House. As her Majesty came out in her carriage, the moment it came in front of me, the carriage stopped for a second, and the prisoner made a step in advance and struck her Majesty."

Sir James Clark said—"I am physician to her Majesty. I was sent for to see her Majesty on the evening in question, and arrived at the Palace between eight and nine o'clock. I examined her forehead, and found a considerable tumour on the outer angle of the right brow, and a small cut. It had been bleeding, but the blood had stopped. I was surprised to see so much injury done by such a small stick, and I therefore infer it was used very violently. Her Majesty's bonnet was cut through. I think the skin was cut by the stick, and not by the wire margin of the bonnet."

This was the case for the prosecution.

Mr. Cockburn then addressed the Jury for the prisoner. He said if the Attorney-General, appearing for the prosecution, felt so deeply the painful nature of the case they were now called upon to investigate, they might very well imagine how much more painful was the duty which he, as the counsel for the prisoner, was called upon to perform. An outrage had been committed upon a Sovereign who was revered and loved by all classes of her subjects—upon a Sovereign who, perhaps, enjoyed the love of those subjects more than any other who had ever filled the throne of these realms; and he had to defend an unfortunate gentleman who was charged with the commission of that outrage. His learned friend had truly anticipated the nature of the defence he should offer. He would not attempt to trifle with the understanding of the jury by an endeavour to deny that the prisoner had actually committed the act with which he was charged, but he trusted to be able to satisfy them that, at the time he committed it, he was in such a state as not to render him responsible. What motive could there be for the commission of the offence? As to any traitorous design, that was quite out of the question. A man did not attempt to carry out traitorous views of that character with a small cane such as the one that had been produced. Was there any political motive? Nothing of the sort could be attributed to this unfortunate gentleman. To what, then, could this act by possibility be referred but to the sudden impulse of a disordered mind? Since the proceeding, the prisoner had been asked how he came to commit the act, and he was unable to give the least explanation; all he could say was, that the act was the result of a momentary impulse which he was unable to control.

The following witnesses were then examined for the defence:—

Colonel John Vandeleur said—"I was lieutenant-colonel of the 10th Hussars when Mr. Pate joined the regiment in the year 1841 as cornet. He afterwards became lieutenant. He remained in the regiment till the month of March, 1846. While we were stationed at Cahir, I remember an accident happening to the prisoner's horses and dog. From the moment the prisoner joined the regiment I thought there was something strange in his conduct. His hair was cut very short, and I fancied his head had been shaved. He discharged his duties as an officer very well; and as to his being a gentleman there is no doubt about that. He was a person of mild demeanour, and very much respected in the regiment. He had three horses and a Newfoundland dog, and he was very much attached to them. The prisoner's horses and dog were bitten by a mad dog belonging to another officer, and they were all destroyed. From this period I observed a great change in his conduct, and he appeared very much excited in consequence of a correspondence that took place between his father and the Duke of Wellington upon the subject of these horses. A claim was made upon Captain Wallington, to whom the dog that bit the prisoner's horses belonged, through the Duke of Wellington, and the prisoner seemed hurt that his friends should have made such a claim. He appeared to avoid company, and used to take long, solitary walks by himself, and he complained to me that he was ill just before he returned to England. He said he had applied to the doctor of the regiment, and he could give him no relief. I asked him what was the matter with him, and he said his stomach and bowels were full of bricks, and that the doctor had not the skill to remove them. To the best of my knowledge the prisoner never replaced the horses that were killed, except one. The prisoner was constantly on the sick list after this. I considered he was labouring under a delusion. I sent him in command of a detachment from Newbridge to Dublin in 1845, and he had orders to return the next day, but he left his detachment at Dublin without leave, and returned to England. When he came back he appeared very well, and he gave no explanation for his going away. I communicated with his father in as delicate a manner as I could, and the prisoner left the regiment two months afterwards."

Captain Frith said—"Prisoner has also told me that there were stones and bricks in his stomach. Sometimes he was very reserved, and at others very wild and excited, without any apparent cause, and I thought his mind was impaired by the loss of his horses and dog. When he left the regiment he gave all his appointments to the adjutant of the regiment, which was not a usual thing. They were very valuable."

Thomas Venn said—"I am a corporal in the 10th Hussars, and was in the regiment when Mr. Pate joined. I remember it being discovered that his horses had been bitten, and they went mad, and two of them were shot. Mr. Pate was very much concerned at the loss of one of these horses. After the first horse was taken ill, he said, that if anything happened to the other—his big horse, as he called him—he did not know what he should do, and he should be inclined to make a hole in the river."

George Pitt, a Serjeant in the 10th Hussars, also spoke to the fact of the demeanour of the prisoner when his horses were destroyed. After they were killed he appeared very much depressed, and his conduct was very eccentric.

Thomas Martin, trumpeter to the regiment, gave similar evidence.

Mr. Robert Francis Pate said—"The prisoner is my son. I remember his leaving the regiment in Ireland without leave. He came down to my residence at Wisbeach, and I ascertained he had not got leave of absence. I told him I was astonished and hurt at his conduct, and asked for an explanation, and he said he had been hunted about Dublin streets by people, and he had seen the same people at the barracks, and he had even seen them about the hotels in London, and he said he had made his escape from Dublin in a vessel coming to Liverpool. I told him I could not let him remain with me, and that he must return immediately to his regiment, and he promised to go back the next morning. The prisoner did go away, and rejoined his regiment, and I afterwards received a letter from his Colonel, advising me to take him out of the regiment. He had leave of absence afterwards, and I met him in London, and he then sold his commission, without my leave or knowledge. I understood from the prisoner that, after paying his debts, he had £1,200 left. Application was afterwards made to me by persons to whom he was indebted, and I went up to London and saw the prisoner, and his appearance was so extraordinary that I was alarmed at it, and consulted Dr. Conolly, and he thought that the presence of the prisoner's sister might make him more comfortable, and advised that any treatment should be postponed for the present.

Charles Dodman said, that he was servant to the prisoner while he was in the 10th Hussars. His conduct was always strange and eccentric. By the Attorney-General—He paid his bills very regularly, and kept the receipts and put them away. Re-examined—He used to shout and sing and whistle in a very extraordinary manner, and the people of the house used to observe upon his conduct.

Edward Lee, a cab-driver, said, he was in the habit of driving the prisoner from November, 1847, and he fetched him regularly every day at one time—a quarter past three. They always went the same route, over Putney-bridge to Putney-heath, and to one particular spot. The prisoner used to get out of the cab and walk through the thickest of the furze bushes and the gorse, and he was out of his sight for about ten minutes. Used to meet him again at one particular spot near a pond, and had seen him stand and look at the pond a few minutes and then jump into the cab. Sometimes the prisoner would tell him to gallop, and then he would pull him up and make him go at a foot pace. They used then to go to a particular place at Barnes-common, where he got out again and walked through all the furze-bushes, and then they went home by Hammersmith-bridge. Witness always thought he was not right in his mind, and in the winter time he was alarmed at him. In all weathers, rain, hail, or snow, he used to get out and walk through the furze-bushes, and he did so when it was quite dark.

Mr. James Starten deposed, that he was a surgeon, residing in Saville-row. He had conversed with the prisoner, and, although there was certainly nothing insane in his conversation, yet, from his mode of talking, he should not set him down as a man possessing a sound mind.

Dr. Conolly, examined by Mr. Cockburn.—"I am the head physician of the Hanwell Lunatic Asylum, and have paid great attention to the malady of insanity. I have conversed with the prisoner since this transaction, and in my opinion he is a person of unsound mind. I form this opinion from the conversations I myself have had with him." By the Attorney-General—"I am not aware that he suffers from any particular delusion. He is well aware that he has done wrong, and regrets it."

Dr. Munro said—"I have had five interviews with Mr. Pate since this transaction, and from my own observation, and what I have heard to-day, I believe him to be of unsound mind. I agree with Dr. Conolly that he is not labouring under any specific delusion." By Mr. Cockburn.—"From all I have heard to-day, and from my personal observation, I am satisfied the prisoner is of unsound mind." Baron Alderson.—"Be so good, Dr. Monro, as not to take upon yourself the functions of the judge and the jury. If you can give us the results of your scientific knowledge upon the point we shall be glad to hear you; but while I am sitting upon the bench I will not permit any medical witness to usurp the functions both of the judge and the jury."*

This closed the case for the prisoner.

The Attorney-General then made a brief and eloquent reply. What was the prisoner's conduct when he was taken into custody? Did it not clearly show that he was perfectly well aware of what he had been doing, and was his conduct anything like that of an insane person? All he endeavoured to do was to palliate his offence. He was perfectly aware he had done wrong, and he sought to extenuate the act by saying that the witnesses could not tell whether he struck at the Queen's face or at her bonnet, and he subsequently said that it was only a little blow with a light stick. This plainly showed that he knew well what he had done, and that it was a wrong act, and it put an end to the defence altogether.

Mr. Baron Alderson then summed up. He said they would have no difficulty with regard to the fact of the prisoner having struck her Majesty, or that his intention was one of those mentioned in the indictment. That he intended to injure her Majesty was apparent from the fact that he actually did injure her, and that blood flowed in consequence of the blow. With regard to alarming her Majesty, probably from the natural courage of the family to which she belonged, that was not done; but there was no doubt that the former count, and also the one charging an intention to break the public peace, had been clearly made out. The Learned Judge then read over the whole of the evidence for the defence, commenting upon it as he proceeded. He went on to say that the prisoner was an object of commiseration was quite clear; and that he should also have been taken better care of, was equally true; but the question they had here to decide was, were they satisfied that he was suffering from a disease of the mind which rendered him incapable of judging whether the act he committed towards the Queen was a right or a wrong act for him to do? If they were not satisfied of this fact, they must say that he was guilty; but, on the contrary, if they thought he was not aware what he was about, or not capable of distinguishing between right and wrong, they would then say that he was not guilty on the ground of insanity.

The Jury retired at twenty minutes past three, and did not return into Court until five minutes past seven, when they gave a verdict of *Guilty*.

The prisoner was immediately called up for judgment.

Baron Alderson addressed him to the following effect:—"Robert Pate, the Jury have found you guilty after a very long and patient inquiry, and there can be no

* The reprimand of the Judge was uncalled for, and unjustifiable and undignified. Dr. Monro was only answering the question put to him by the counsel.

reasonable doubt that they have come to a right conclusion. At the same time, it is clear that you are a person of very eccentric habits, and in some degree differing from other men, and it is probable that it has pleased God to visit you with some mental affliction, for which you are to be pitied. The offence you have committed, however, is one of a very serious and important character. You have been found guilty of striking a woman, which for a soldier is a very shocking thing; but when it is considered that this woman was your sovereign—that it was a lady entitled to the respect of the whole country by her virtues and her exalted position, that act which in an ordinary case would be a very serious offence, under these circumstances becomes truly heinous. I think the Jury were quite right, upon the evidence that was adduced, in not acquitting you upon the ground of insanity. Under all the circumstances the sentence that I feel it my duty to pronounce upon you is, that you be transported beyond the seas for the term of seven years."

The prisoner heard the sentence without betraying the slightest emotion, and when the Learned Judge had concluded his address he bowed to the Court, and immediately turned round and without uttering a word retired to the gaol.

The trial lasted nearly nine hours.

---

